# Supplementary material for: Bottom Contact Metal Oxide Interface Modification Improving the Efficiency of Organic Light Emitting Diodes
Source: Materials (Basel). 2020 Nov 11;13(22):5082. doi: 10.3390/ma13225082 (PMC7696400; doi:10.3390/ma13225082)
Supplement: Supplementary file 1 [file materials-13-05082-s001.zip › materials-979013-supplementary.docx]

**Supplementary Material**

Bottom Contact Metal Oxide Interface Modification Improving the Efficiency of Organic Light Emitting Diodes

Sergey M. Pozov ^1^, Apostolos Ioakeimidis ^1^, Ioannis T. Papadas ^1^, Chen Sun ^2^,
Alexandra Z. Chrusou ^1^, Donal D. C. Bradley ^2,3^ and Stelios A. Choulis ^1,^*

^1^ Department of Mechanical Engineering and Materials Science and Engineering, Molecular Electronics and Photonics Research Unit, Cyprus University of Technology, Limassol 3603, Cyprus; sergey.pozov@cut.ac.cy (S.M.P.); a.ioakeimidis@cut.ac.cy (A.I.); ioannis.papadas@cut.ac.cy (I.T.P.); az.chrusou@cut.ac.cy (A.Z.C.)

^2^ Department of Physics, Clarendon Laboratory, University of Oxford, Parks Rd, Oxford OX1 3PU, UK; chen.sun@physics.ox.ac.uk (C.S.); donal.bradley@kaust.edu.sa (D.D.C.B.)

^3^ Physical Science and Engineering Division, King Abdullah University of Science and Technology (KAUST), Thuwal 23955-6900, Saudi Arabia

***** Correspondence: stelios.choulis@cut.ac.cy

Received: 11 October 2020; Accepted: 9 November 2020; Published: date


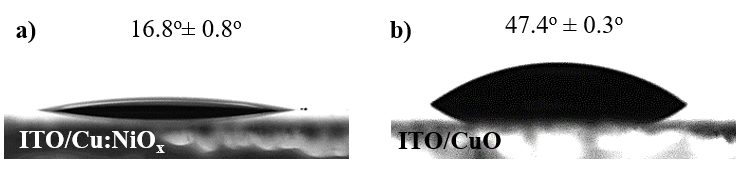


**Figure S1.** Static contact angle of deionized water droplet images on solution combustion synthesized (SCS) metal oxide substrates, a) ITO/Cu:NiO_x_ and b) ITO/CuO_x_.

**Table S1.** Statistical analysis of electroluminescence performance of lab-scale 9 mm^2^ organic light emitting (OLED) devices investigating the effect of spinel film thickness. Statistic sample size of 8 different devices was used to present average values, together with standard deviation, and maximum values in brackets.

| **Anode Electrode** | **Turn on voltage**  **(at ~10 cd/m^2^)**  **[V]** | **Max.**  **Luminance**  **[cd/m^2^]** | **Max. Current**  **Efficiency**  **[cd/A]** | **Max. Power**  **Efficacy**  **[lm/W]** |
| --- | --- | --- | --- | --- |
| ITO/PEDOT:PSS | 2.6 | 9.0k ± 0.2k | 4.9 ± 0.1 (5.0) | 4.4 ± 0.1 (4.5) |
| ITO/(Cu-Li):NiCo_2_O_4_ (~30 nm) | 2.7 | 9.0k ± 0.3k | 3.00 ± 0.4 (3.4) | 2.5 ± 0.5 (2.8) |
| ITO/(Cu-Li):NiCo_2_O_4_ (~20 nm) | 2.7 | 8.9k ± 0.2k | 3.2 ± 0.1 (3.4) | 2.5 ± 0.3 (2.9) |
| ITO/(Cu-Li):NiCo_2_O_4_ (~15 nm) | 2.7 | 9.0k ± 0.5k | 3.5 ± 0.1 (3.7) | 2.9 ± 0.2 (3.1) |
| ITO/(Cu-Li):NiCo_2_O_4_ (~10 nm) | 2.8 | 8.0k ± 1.5k | 3.4 ± 0.3 (3.7) | 2.8 ± 0.3 (3.0) |


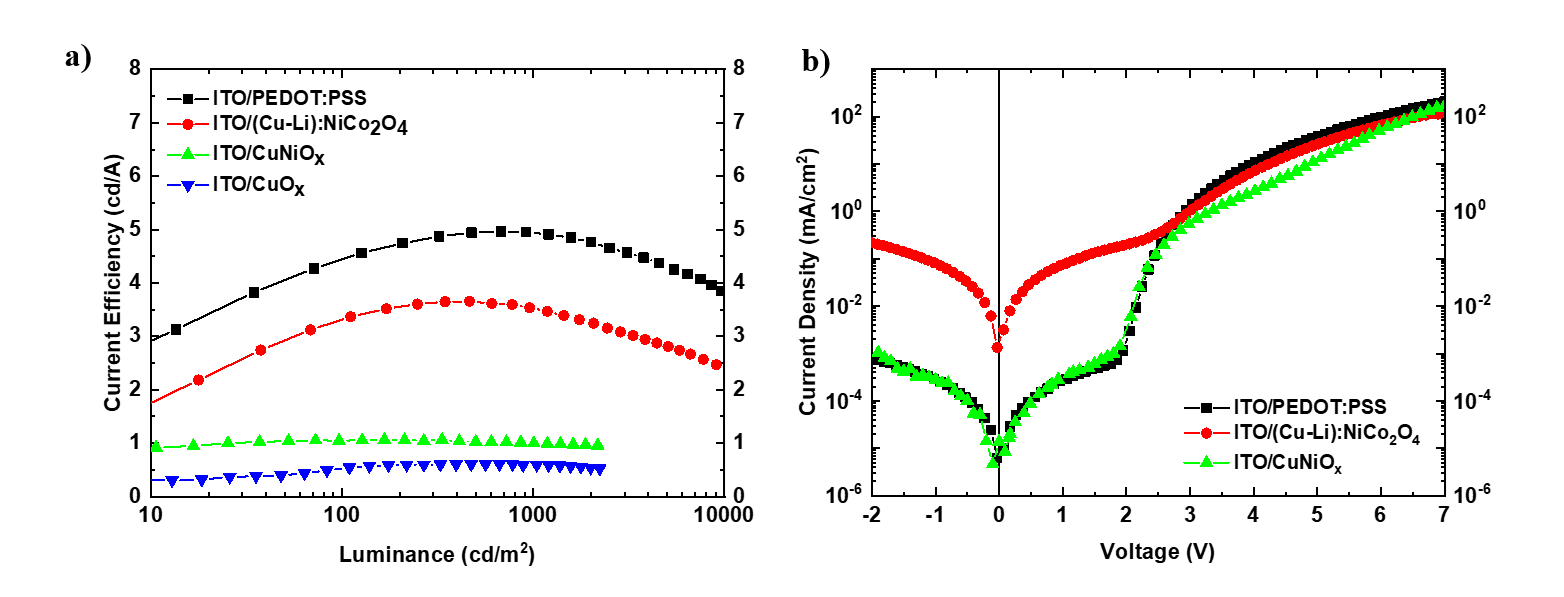


**Figure S2.** Performance of OLED devices based on ITO/SCS metal-oxide bottom electrodes, using different metal oxide interlayers, a) current efficiency over device luminance, b) semi-log *JV* - leakage current characteristics.

Replacing (Cu-Li):NiCo_2_O_4_ by Cu:NiO_x_ or CuO_x_ using a type B (indium tin oxide/metal oxide) device configuration, the performance analysis in Figure S2 points out to even lower efficiencies. Both Cu:NiO_x_ and CuO_x_ showed reduced performance in comparison to the optimized (Cu-Li):NiCo_2_O_4_, due to their much lower electrical conductivity Cu:NiO_x_ (~1.25 x 10^-3^ S/cm) [1] and CuO_x_ (~1 x 10^-6^ S/cm) [2], in comparison to (Cu-Li):NiCo_2_O_4_ (~4 S/cm) [3]. Studying the Figure S2b Cu:NiO_x_ presents similar leakage current characteristics to our reference device ITO/PEDOT:PSS due to its high LUMO level (~1.8 eV) of [4] that ensures electron blocking from super-yellow active layer, however the much lower forward bias current lead to reduced device efficiency. The effect of proper energy level alignment and electrical conductivity of MO, is strongly distinct in CuO_x_ with much deeper HOMO ~5.6 eV [5], strongly resisted the charge injection resulting in non-reproducible JVs, and significantly reduced device performance as shown in Figure S2a. Based on the fact that the low conductive PEDOT:PSS AI4083 formulation, used in this work has an electrical conductivity in the range of 10^-3^ – 10^-4^ S/cm, which is much lower that (Cu-Li):NiCo_2_O_4_ (~4 S/cm) we conclude that electrical conductivity plays a role among the examined MOs, however the proper energy level alignment with the active layer polymer is the most dominant parameter that affects the performance of OLED devices.


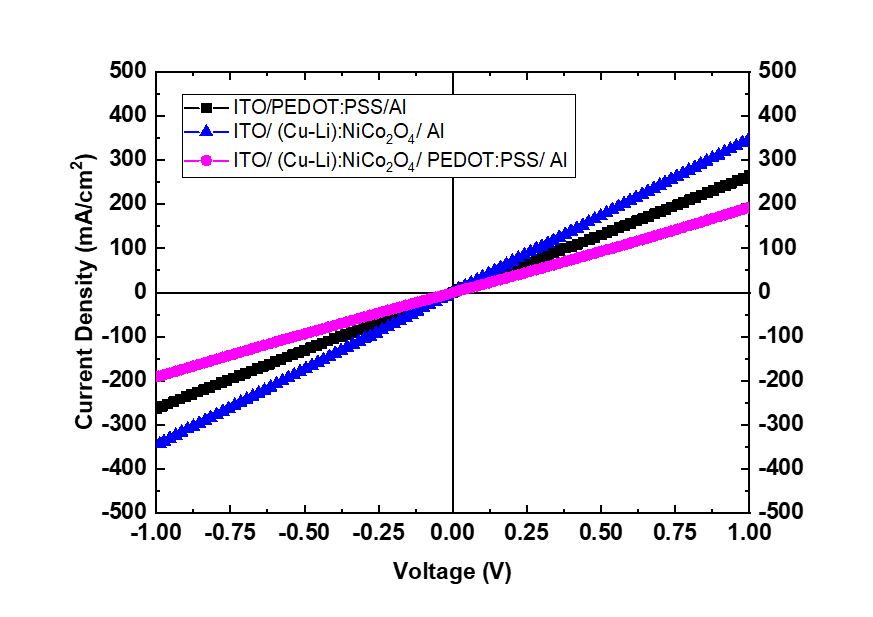


**Figure S 3.** Current density – voltage (JV) characteristics of PEDOT:PSS, (Cu-Li):NiCo_2_O_4_ and (Cu-Li):NiCo_2_O_4_/PEDOT:PSS, sandwiched between ITO anode and Al cathode electrodes.


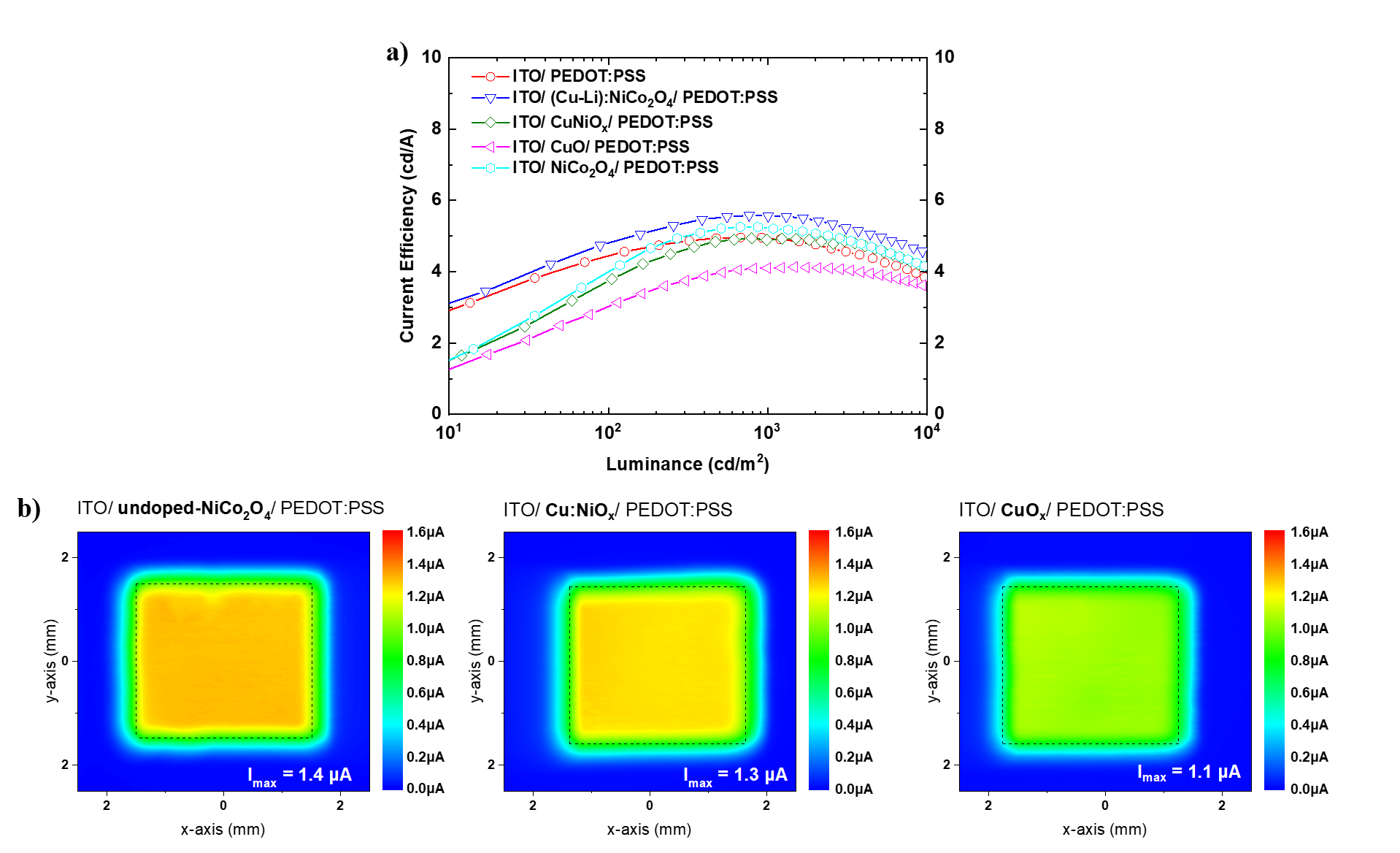


**Figure S4**. Performance of OLED devices based on ITO/SCS metal-oxide/ PEDOT:PSS bottom electrodes, using different metal oxide interlayers, a) current efficiency *versus* device luminance, b) photocurrent mapping images of complete 3 x 3 mm^2^ SY OLED devices.

Device current efficiency presented in Figure S4a, follows the order, from highest to lowest, of ITO/undoped-NiCo_2_O_4_/PEDOT:PSS, followed by ITO/Cu:NiO_x_/PEDOT:PSS and last ITO/CuO_x_/PEDOT:PSS bottom electrode. The same trend was also observed in the generated photocurrent images Figure S4b, and the wetting properties of the examined SCS metal oxides (see Figure 1 and Figure S1).

[1] J.W. Jung, C.C. Chueh, A.K.Y. Jen, A Low-Temperature, Solution-Processable, Cu-Doped Nickel Oxide Hole-Transporting Layer via the Combustion Method for High-Performance Thin-Film Perovskite Solar Cells, Adv. Mater. 27 (2015) 7874–7880. https://doi.org/10.1002/adma.201503298.

[2] D.M. Jundale, P.B. Joshi, S. Sen, V.B. Patil, Nanocrystalline CuO thin films: Synthesis, microstructural and optoelectronic properties, J. Mater. Sci. Mater. Electron. 23 (2012) 1492–1499. https://doi.org/10.1007/s10854-011-0616-2.

[3] A. Ioakeimidis, I.T. Papadas, D. Tsikritzis, G.S. Armatas, S. Kennou, S.A. Choulis, Enhanced photovoltaic performance of perovskite solar cells by Co-doped spinel nickel cobaltite hole transporting layer, APL Mater. 7 (2019) 021101. https://doi.org/10.1063/1.5079954.

[4] M. Kim, C.W. Joo, J.H. Kim, W. Choi, J. Lee, D. Lee, H. Cho, H. Lee, S. Park, N.S. Cho, H. Cho, C.W. Lee, D.Y. Jeon, B.H. Kwon, Conductivity Enhancement of Nickel Oxide by Copper Cation Codoping for Hybrid Organic-Inorganic Light-Emitting Diodes, ACS Photonics. 5 (2018) 3389–3398. https://doi.org/10.1021/acsphotonics.8b00656.

[5] J.D. Kwon, S.H. Kwon, T.H. Jung, K.S. Nam, K.B. Chung, D.H. Kim, J.S. Park, Controlled growth and properties of p-type cuprous oxide films by plasma-enhanced atomic layer deposition at low temperature, Appl. Surf. Sci. 285 (2013) 373–379. https://doi.org/10.1016/j.apsusc.2013.08.063.
